# Efficacy of adalimumab in refractory juvenile idiopathic arthritis related uveitis: report of 6 cases

**DOI:** 10.1186/1546-0096-9-S1-P180

**Published:** 2011-09-14

**Authors:** O Vougiouka, E Atsali, C Panousi-Vlahou, S Azaris, A Koidou

**Affiliations:** 1 2nd Department of Paediatrics, Athens University Medical School - Paediatric Rheumatology Outpatient Clinic, Ophthalmology Department,“P. A. Kyriakou” Children’s Hospital, Greece; 2 3rd Department of Paediatrics, University of Athens , "Attikon" Hospital , Greece

## Background

Chronic anterior uveitis is the most common ocular complication of Juvenile Idiopathic Arthritis (JIA) predominantly affecting young girls with early onset oligo-JIA and seropositive antinuclear antibodies (ANA).

## Aim

We retrospectively evaluated the efficacy of adalimumab in 6 children with refractory JIA related uveitis.

## Methods

We analyzed 6 children (5 girls/1 boy) with JIA (5 persistent oligo-, 1 extended oligo- JIA) and chronic uveitis. All children were ANA seropositive. Uveitis was bilateral in 5 children and appeared in half of the children after the presentation of arthritis, in 2 children concomitantly with arthritis and in 1 child before arthritis.

## Results

Ocular symptoms were unresponsive to treatment with local and systemic steroids, methotrexate plus cyclosporine in all patients. One child had also received etanercept for 2.5 years with poor response. Due to frequent relapses of uveitis (especially when tapering local steroids) we commenced treatment with adalimumab (24mg/m^2^ of body surface sc/14 days), while cyclosporine was withdrawn. The mean duration of uveitis until then was 27 months and the mean age of patients at the initiation of treatment was 7.3 years. All patients had at least one complication of uveitis (synechiae: 2 children, band keratopathy: 2 children, cystoid macular edema: 2 children, cataract: 1 child, glaucoma: 2 children). The mean duration of adalimumab treatment was 4.5 months. No side effects were observed. During adalimumab treatment uveitis subsided in all patients with no recurrence neither requiring local treatment. Methotrexate was reduced to the lower dose of 7.5 mg/wk.

## Conclusions

Adalimumab, a fully humanized anti-TNF antibody, appears to be a promising, effective and safe agent in patients with refractory JIA related uveitis.

